# microRNA-92a regulates the expression of aphid bacteriocyte-specific secreted protein 1

**DOI:** 10.1186/s13104-019-4665-6

**Published:** 2019-09-30

**Authors:** Honglin Feng, Joun S. Park, R. Grace Zhai, Alexandra C. C. Wilson

**Affiliations:** 1 Department of Biology, University of Miami, Coral Gables, FL 33146 USA; 2 Department of Molecular and Cellular Pharmacology, Miller School of Medicine, University of Miami, Miami, FL 33136 USA; 3 Present Address: Boyce Thompson Institute, Ithaca, NY 14853 USA

**Keywords:** Aphid, miRNA, Symbiosis, Bacteriocyte, SP1, Dual luciferase assay

## Abstract

**Objective:**

Aphids harbor a nutritional obligate endosymbiont in specialized cells called bacteriocytes, which aggregate to form an organ known as the bacteriome. Aphid bacteriomes display distinct gene expression profiles that facilitate the symbiotic relationship. Currently, the mechanisms that regulate these patterns of gene expression are unknown. Recently using computational pipelines, we identified miRNAs that are conserved in expression in the bacteriomes of two aphid species and proposed that they function as important regulators of bacteriocyte gene expression. Here using a dual luciferase assay in mouse NIH/3T3 cell culture, we aimed to experimentally validate the computationally predicted interaction between *Myzus persicae* miR-92a and the predicted target region of *M. persicae* bacteriocyte-specific secreted protein 1 (SP1) mRNA.

**Results:**

In the dual luciferase assay, miR-92a interacted with the *SP1* target region resulting in a significant downregulation of the luciferase signal. Our results demonstrate that miR-92a interacts with *SP1* to alter expression in a heterologous expression system, thereby supporting our earlier assertion that miRNAs are regulators of the aphid/*Buchnera* symbiotic interaction.

## Introduction

Aphids are obligately dependent on their ancient endosymbiotic relationship with the gamma-proteobacterium *Buchnera aphidicola* [1, 2]. The symbiont, *Buchnera*, is housed in a specialized organ called the bacteriome, inside specialized host cells called bacteriocytes [1–4]. Bacteriomes are enriched in expression of genes associated with functions that include amino acid biosynthesis and metabolism, and transporters that mediate metabolite exchange between aphid and *Buchnera* [5–8]. Bacteriome gene expression profiles also feature expression of two groups of aphid orphan genes: bacteriocyte-specific cysteine-rich proteins and aphid-specific putative secreted proteins [9]. One putative secreted protein is secreted protein 1 (SP1), a gene whose expression is restricted to bacteriocytes. The lineage specificity of SP1, coupled with its tissue-specific expression suggests that this orphan gene may have contributed to the evolution of aphid-specific traits, i.e. the symbiosis with *Buchnera* [9].

**Fig. 1:**
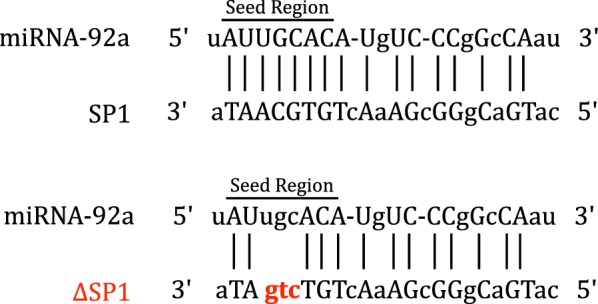
Mpe-miR-92a is predicted to regulate secreted protein 1 (SP1). The predicted base pairing between mpe-miR-92a and the target region in the 3′ UTR of the SP1 transcript. The seed regions of mpe-miR-92a are indicated; the mutated target regions are highlighted in red

Recently using two aphid species, the pea aphid, *Acyrthosiphon pisum*, and the green peach aphid, *Myzus persicae*, we identified 14 evolutionary conserved microRNAs (miRNAs) that were bacteriome-specific and/or bacteriome-enriched and were predicted to regulate 103 aphid genes, many of which have known importance to the aphid/*Buchnera* symbiosis [10]. Among those predictions, miR-92a was significantly upregulated in bacteriocytes and predicted to target the bacteriocyte-specific SP1 (Fig. 1) [10]. Remarkably, miR-92a has been shown to be important in a great diversity of host/microbe interactions that include host/virus interactions in a mosquito [11] and a fall armyworm [12], and host/pathogen interactions in a mosquito [13], marine filter feeders [14, 15], a spider mite [16], and a fish [17]. Here, we experimentally interrogate our computationally predicted interaction of *M. persicae* mpe-miR-92a with *SP1*.

## Main text

### Methods

#### Target prediction

In our previous study we used miRanda [18], PITA [19] and RNAhybrid [20] to predict potential miRNA::mRNA interactions [10]. All three algorithms predicted the same seed region. The target site predicted by miRanda was the largest and spanned the target sites predicted by PITA and RNAhybrid (Additional file 1: Figure S1), thus, we designed our dual luciferase assay using the miRanda target site prediction.

#### *Mus musculus* embryonic fibroblast NIH/3T3 cell culture

We maintained NIH/3T3 cells under sterile conditions at 37 °C with 5% CO_2_. Cells were cultured in the ATCC-formulated Dulbecco’s Modified Eagle’s Medium with bovine calf serum (Gibco, USA) at 10%, gentamycin at 0.5% (v/v) and penicillin–streptomycin at 1% (v/v).

#### Plasmid preparation and miRNA mimic synthesis

To validate the predicted miRNA::mRNA interaction, we utilized pmirGLO Dual-Luciferase miRNA target expression vector (pmirGLO) (Promega, USA) and miRNA mimics. The pmirGLO vector expresses two luciferases: the firefly luciferase (an experimental reporter that can be subject to the effect of miRNA regulation) and the *Renilla* luciferase (an internal control). Using pmirGLO, we prepared an experimental plasmid, a negative and a positive control plasmid. The experimental plasmid, pmirGLO-SP1, contained a synthesized miR-92a::*SP1* target region corresponding to the miR-92a binding site on the *SP1* 3′ UTR of *M. persicae* (Additional file 2: Table S1) [10]. The negative control plasmid, pmirGLO-ΔSP1, contained a synthesized mutated *SP1* (ΔSP1) that was designed based on the *M. persicae* miR-92a::*SP1* target region using the Illegitimate microRNA predictor (Additional file 2: Table S1) [21]. We obtained the positive control plasmid, pmirGLO-miR21T, that includes the *M. musculu*s miR-21 target site from Promega, USA. Our experiments used two miRNA mimics, a miR-92a mimic (Fig. 1) and a non-specific negative control siRNA i.e. AllStars Negative Control siRNA from QIAGEN, USA (Cat#: SI03650318). AllStars Negative Control siRNA has a proprietary sequence with no homology to any known mammalian gene.

#### Cell transfection

We performed the transient cell transfection experiment three times. For the first two experiments, we used the Effectene Transfection Reagent (Qiagen, USA). Briefly, 400 ng DNA plasmid and/or 300 nM miRNA mimics were used to transfect/co-transfect 4 × 10^5^ cells/well in 6-well plates for 24 h. Then, we harvested cells at 48 h for the dual luciferase assay. In the third experiment, we used the Attractene Transfection Reagent (Qiagen, USA). Briefly, 400 ng DNA plasmids and/or 6 pmol miRNA mimics were used to transfect/co-transfect 1.6 × 10^5^ cells/well in 24-well plates. Cells were transfected for 48 h and harvested for the dual luciferase assay.

#### Dual luciferase assay

Transfected cells were assayed using the Dual-GLO^**®**^ Luciferase Assay System (Promega, USA). For each sample, the firefly and *Renilla* luciferase activities were measured sequentially by collecting emitted luminescence from the entire visible spectrum (300–700 nm) on a Synergy H1 Multi-Mode Reader (BioTek, USA). Briefly, the firefly luciferase activity was measured 10 min after induction of cell lysis and provision of the firefly luciferase substrate. Then, we quenched the firefly luciferase reaction and provided the *Renilla* luciferase substrate. Ten minutes later we captured the *Renilla* luciferase activity. The luminescence measurement for each well represents the average of 12 serial luminescence readings.

Each plate included four technical replicates of each treatment, plus four control technical replicates (cells were exposed only to the transfection reagents) to allow background luminescence subtraction. Following background subtraction, we calculated the ratios of firefly/*Renilla* luminescences. To compare data across the three experiments we normalized data within each experiment to the empty pmirGLO control treatment by dividing each firefly/*Renilla* ratio by the mean firefly/*Renilla* ratio of the empty pmirGLO control treatment.

#### Statistical analyses

We tested for differences in the normalized firefly/*Renilla* ratios among treatments using one-way ANOVA with a fixed factor of treatment and a block effect of experiment, followed by a Tukey HSD post hoc test for multiple comparisons in SPSS v.24.

### Results

#### miR-92a interacts with the predicted target region of SP1 mRNA in NIH/3T3 cells

**Fig. 2:**
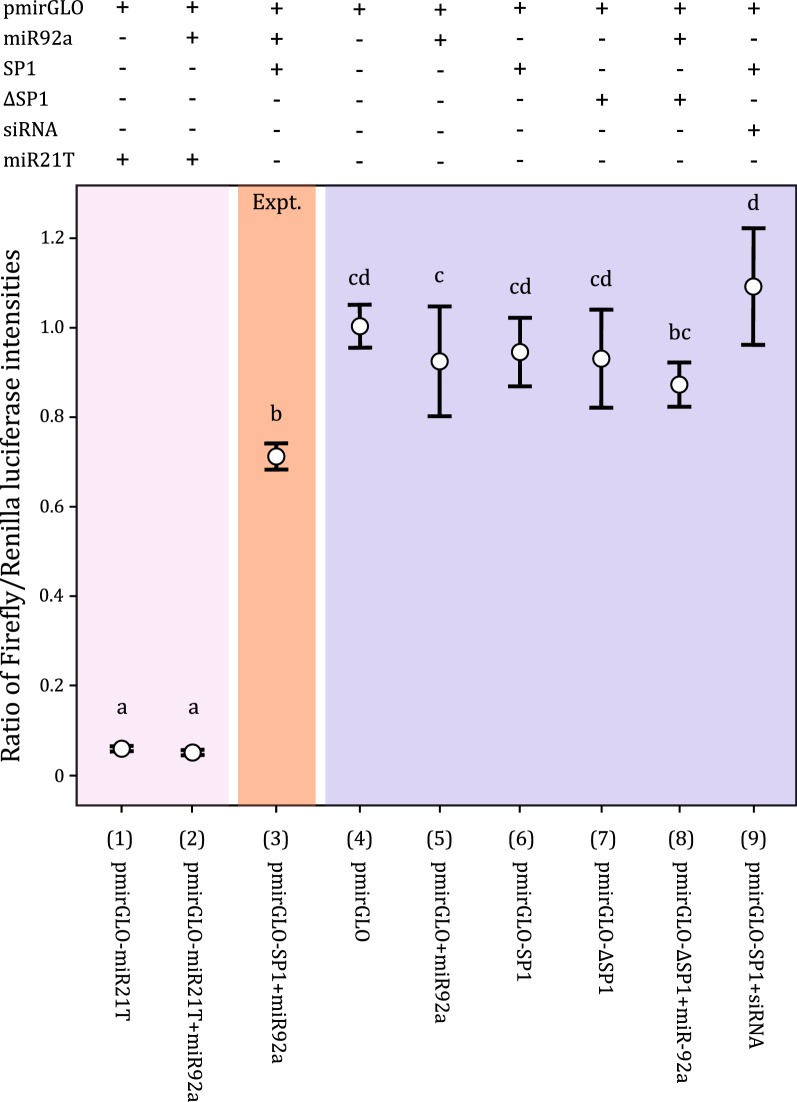
Dual luciferase assay of mpe-miR-92a::*SP1* interactions. The ratios of the firefly luciferase versus the *Renilla* luciferase activities were compared across different treatments. “+” means the presence of the element in the treatment and “−” means the absence of the element in the treatment. Expt.: Experimental treatment. The data was tested using 1-way ANOVA, controlling for random block effects, followed by Tukey HSD post hoc analysis. The lowercase letters above each whisker (a, b, bc, c, cd, d) denote statistically significant differences between treatments. Error bar = ± standard error (n = 12) from three experimental replicates

To test the predicted miR-92a::*SP1* interaction, we performed a dual luciferase assay in NIH/3T3 cells that we transfected with a pmirGLO-SP1 construct together with mature miR-92a (pmirGLO-SP1 + miR-92a, treatment 3 in Fig. 2). In parallel we performed a series of controls that included (i) cells transfected with an NIH/3T3 endogenous miRNA construct: pmirGLO-miR21T (treatment 1, Fig. 2), (ii) a pmirGLO-miR21T construct + miR-92a (treatment 2, Fig. 2), (iii) a pmirGLO empty construct (treatment 4, Fig. 2), (iv) a pmirGLO empty construct + miR-92a (treatment 5, Fig. 2), (v) a pmirGLO-SP1 construct (treatment 6, Fig. 2), (vi) a pmirGLO-ΔSP1 construct (treatment 7, Fig. 2), (vii) a pmirGLO-ΔSP1 construct + miR-92a (treatment 8, Fig. 2), and (viii) a pmirGLO-SP1 construct + siRNA (treatment 9, Fig. 2).

In the dual luciferase assay, we validated that mpe-miR-92a specifically interacts with the predicted *SP1* target region. After we removed any random block effects (Table 1: experiment, F(2) = 0.948, *p* = 0.391), we observed significant differences in firefly/*Renilla* ratios between groups under different treatment conditions (Table 1: treatment, F(8, 11) = 114.567, *p* < 0.0001; Fig. 2). First, the significant difference between the pmirGLO-miR21T treatments and the empty pmirGLO (*p* < 0.0001) indicated that the dual luciferase assay was working properly (Fig. 2, treatments 1 vs 4, 2 vs 4). Second, the treatment of pmirGLO-SP1 + miR-92a was also significantly different from empty pmirGLO (*p* < 0.0001, Fig. 2, treatments 3 vs 4) and pmirGLO-SP1 only (*p* = 0.001, Fig. 2, treatments 3 vs 6), indicating that mpe-miR-92a specifically interacted with the predicted *SP1* target region, resulting in significant downregulation of the luciferase signal. Third, we found no difference between treatments of pmirGLO-SP1 only and empty pmirGLO (*p* = 0.976, Fig. 2, treatments 4 vs 6) indicating an absence of endogenous NIH/3T3 miRNA interactions with the predicted *SP1* target region. Fourth, we observed a significant difference between the pmirGLO-SP1 + miR-92a and the pmirGLO + miR-92a (*p* = 0.003, Fig. 2, treatments 3 vs 5) indicating that the miR-92a downregulation of pmirGLO-SP1 was not the result of interactions between miR-92a and the pmirGLO vector. Fifth, we found no significant difference between the pmirGLO-SP1 + miR-92a and pmirGLO-ΔSP1 + miR-92a treatments (*p* = 0.072, Fig. 2, treatments 3 vs 8), suggesting that miR-92a can interact with ΔSP1. However, we found no significant difference between the pmirGLO-ΔSP1 and pmirGLO-ΔSP1 + miR-92a (*p* = 0.973, Fig. 2, treatments 7 vs 8), suggesting that the interaction between miR-92a and ΔSP1 was not as strong as the interaction between miR-92a and the *bona fide SP1* target region (Fig. 2). We suspect that the interactions between miR-92a and Δ*SP1* may result from (i) possible G-U wobble base-pairings between the mutated nucleotides and miR-92a; and (ii) extensive base-pairing in the non-seed region of miR-92a (Fig. 1), because the non-seed region of miRNAs (nucleotides 12–17) have been shown in mammalian cells to be important for miRNA targeting [22, 23]. Lastly, we found a significant difference in signal between the pmirGLO-SP1 + miR-92a and pmirGLO-SP1 + siRNA (Fig. 2, Treatments 3 vs 9), and no difference in signal between the pmirGLO-SP1 + siRNA, empty pmirGLO, and pmirGLO-SP1 treatments (Fig. 2, Treatments 4 vs 6; 4 vs 9; 6 vs 9), suggesting that the interaction between miR-92a and *SP1* is sequence specific.

**Table 1:** ANOVA statistics of mpe-miR-92a and *SP1* dual luciferase assays

Source	Type III Sum of squares	df	Mean square	F	Sig.
Corrected model	15.156^a^	10	1.516	91.843	0.000
Intercept	57.933	1	57.933	3510.743	0.000
Treatment	15.124	8	1.891	114.567	0.000
Experiment	0.031	2	0.016	0.948	0.391
Error	1.601	97	0.017		
Total	74.690	108			
Corrected total	16.756	107			

^a^R Squared = 0.904 (Adjusted R Squared = 0.895)

### Discussion

#### Aphid miR-92a interacts with SP1

In our dual luciferase assay in NIH/3T3 cells, mpe-miR-92a physically interacted with the predicted *SP1* target region, resulting in significant downregulation of gene expression (Fig. 2). This assay validates our earlier computational prediction that *SP1* is regulated by miR-92a [10] and further, highlights both the role of miRNAs as regulators of gene expression in aphid bacteriomes, and the potential of targeting the miR-92a::*SP1* interaction for pest aphid control [24, 25].

#### miRNAs regulate gene expression in aphid bacteriocytes

Aphid gene expression in bacteriomes is crucial to the function of the aphid/*Buchnera* symbiosis. What remains elusive are the mechanisms by which the expression of these bacteriocyte-specific genes are regulated.

The abundance of proteins in a cell results from the dynamic interplay of transcriptional, post-transcriptional (e.g. miRNA regulation), translational, and post-translational regulation [26–28]. The identification of transcriptional regulation in aphids has been limited to studies of aphid development and regulation of *Buchnera* gene expression. Three transcription factors: Distal-less, Engrailed, and Ultrabithorax/Abdominal-A have been implicated in bacteriocyte specification and development in aphids [29]. While in *Buchnera*, studies have demonstrated limited transcriptional regulation of the expression of heat shock [30–33] and amino acid biosynthesis genes [34, 35]. More recently, a remarkable example of post-translational regulation of amino acid biosynthesis in bacteriocytes has been proposed in *A. pisum* by glutamine transporter, ApGLNT1 [36]. ApGLNT1 localizes to the bacteriocyte plasma membrane where it transports glutamine from aphid hemolymph into bacteriocyte cells. Importantly, glutamine transport is competitively inhibited by a *Buchnera*-synthesized essential amino acid end-product, arginine. Thus ApGLNT1 regulates the transport of glutamine, a host-supplied amino acid precursor, by an endosymbiont-synthesized end-product via substrate feedback inhibition at the post-translational level [36].

In other work, post-transcriptional regulation of gene expression has been suggested to be important for regulation of the aphid/*Buchnera* endosymbiosis. For example, comparison of *Buchnera* gene expression in embryonic and maternal bacteriocytes found no differences in mRNA abundance, but differences in protein abundance that have been attributed to a *Buchnera* encoded set of conserved small RNAs [37, 38]. In addition, we recently identified a set of evolutionarily conserved aphid miRNAs that are bacteriome-specific and/or bacteriome-enriched in *M. persicae* and *A. pisum*. Notably, many of the conserved miRNAs were predicted to target bacteriocyte-specific genes of known importance to aphid/*Buchnera* symbiosis [10]. Here using a heterologous expression system, we validated one of our predicted miRNA::mRNA interactions, the miR-92a::*SP1* interaction. Our validation of the miR-92a::*SP1* interaction, coupled with our earlier genome-wide analyses, highlight miRNAs as post-transcriptional regulators in the aphid/*Buchnera* symbiosis.

#### miR-92a and its targets are potential targets for aphid control

Recent attempts have been made to develop miRNAs as tools for pest control (reviewed in [25]), either by engineering miRNAs for insecticidal activities [39] or by silencing insect defensive miRNAs [40]. Here we have validated the role of miR-92a in regulation of the orphan, bacteriocyte-expressed gene *SP1*, a gene that encodes a secreted protein that has been argued to be important for symbiotic function. Since aphids lacking a stable *Buchnera* symbiosis fail to reproduce [41–43], it follows that miR-92a offers promise as a target for control of pest aphid populations.

## Limitations

We validated the *M. persicae* miR-92a::*SP1* interaction in a heterologous expression system, the interaction remains elusive in vivo.

## Supplementary information

**Additional file 1: Figure S1.** Prediction of the miR-92a::*SP1* interaction by miRanda, PITA, and RNAhybrid in *M. persicae* (10). The target region predicted by miRanda includes the regions predicted by PITA and RNAhybrid. Thus, the sequence of the miRanda prediction was used to construct the experimental plasmid, pmirGLO-SP1. Grey shaded area marks the seed region of the mature miRNA.

**Additional file 2: Table S1.** Oligonucleotides used for preparing pmirGLO-target plasmids.

## Data Availability

All data generated or analyzed during this study are included in this published article and its supplementary information files.

## Abbreviations

miRNA: microRNA
SP1: secreted protein 1
ΔSP1: mutated secreted protein 1
ApGLNT1: *Acyrthosiphon pisum* glutamine transporter 1
pmirGLO: pmirGLO Dual-Luciferase miRNA target expression vector
