# Supplementary figures and images for: microRNA-92a regulates the expression of aphid bacteriocyte-specific secreted protein 1
Source: BMC Res Notes. 2019 Sep 30;12:638. doi: 10.1186/s13104-019-4665-6 (PMC6767646; doi:10.1186/s13104-019-4665-6)

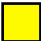 miRanda

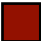 PITA

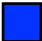 RNAhybrid

miR-92a

5' UAUUGCACA--UgUC--CCGGCCAau 3'

| | | | | | | | | | | | | | | |

*SP1*-3'UTR

3' ATAACGTGTCAaAGCGGGCAGTac 5'

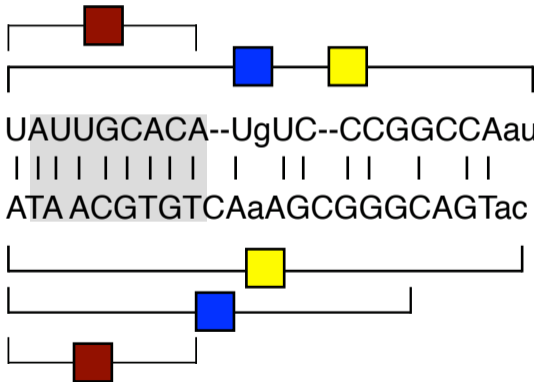

Supplement: Supplementary file 1 — Additional file 1: Figure S1. Prediction of the miR-92a::SP1 interaction by miRanda, PITA, and RNAhybrid in M. persicae (10). The target region predicted by miRanda includes the regions predicted by PITA and RNAhybrid. Thus, the sequence of the miRanda prediction was used to construct the experimental plasmid, pmirGLO-SP1. Grey shaded area marks the seed region of the mature miRNA. [file 13104_2019_4665_MOESM1_ESM.pdf]
