# Supplementary material for: microRNA-92a regulates the expression of aphid bacteriocyte-specific secreted protein 1
Source: BMC Res Notes. 2019 Sep 30;12:638. doi: 10.1186/s13104-019-4665-6 (PMC6767646; doi:10.1186/s13104-019-4665-6)
Supplement: Supplementary file 2 — Additional file 2: Table S1. Oligonucleotides used for preparing pmirGLO-target plasmids. [file 13104_2019_4665_MOESM2_ESM.docx]

**Table S1** Oligonucleotides used for preparing pmirGLO-target plasmids

|  | NheI EcoRI Flank miR-92a target SalI |
| --- | --- |
| SP1 sense: | 5’ CTAGC TA GAATTC TAAT *CATGACGGGCGAAACTGTGCAATA* G 3’ |
| SP1 antisense: | 3’ G AT CTTAAG ATTA *GTACTGCCCGCTTTGACACGTTAT* CAGCT 5’ |
| DSP1 sense: | 5’ CTAGC TA GAATTC TAAT *CATGACGGGCGAAACTGT****ctg****ATA* G 3’ |
| DSP1 antisense: | 3’ G AT CTTAAG ATTA *GTACTGCCCGCTTTGACA****gac****TAT* CAGCT 5’ |
